# XRF online analyzer for measurements of P_2_O_5_ content in phosphate slurry

**DOI:** 10.1038/s41598-023-45181-5

**Published:** 2023-10-20

**Authors:** Ismail Ben Amar, Andrew Thomas, Claus Bachmann, Anass Hafnaoui, Hafid Griguer, Amine Miled, Younès Messaddeq

**Affiliations:** 1 Department of Electrical and Computer Engineering, Université Laval, Quebec City, QC Canada; 2 Digital Innovation Center of Excellence DICE, Mohammed VI Polytechnic University UM6P, Ben Guerir, Morocco; 3 J&C Bachmann GmbH, Pfinztal, Germany; 4 OCP Group S.A, Jorf Lasfar, El Jadida, Morocco; 5 Center for Optics, Photonics, and Lasers, Université Laval, Quebec City, QC Canada

**Keywords:** Sensors, Characterization and analytical techniques, X-rays, Mineralogy, Sedimentology

## Abstract

Online X-ray Fluorescence (XRF) setup was constructed and optimized for analysing the P_2_O_5_ content in phosphate slurry (PS). Serval samples were analysed using two configurations of the setup, one with low and vertical flow and another with high and horizontal flow. The mean absolute error achieved through the first configuration was 0.87% and 0.38% using the second configuration. Reference samples were analyzed using the two configurations to construct the calibration curves. The curves cover a concentration range of P_2_O_5_ from 13.50 to 18.50% when considering the horizontal flow configuration, and a range of 14.00–15.60% when considering the vertical flow setup. An experimental study was conducted in order to optimize the measurement parameters for the online measurement of P_2_O_5_ in the phosphate slurry using the horizontal flow setup. A good signal-to-noise ratio (SNR) of $P\left(K\alpha \right)$ was attained using an excitation energy of 20 kV or 25 kV, an excitation current of 600 µA, a distance of 18 mm between the sample and the detector, a measurement time of 60 s per spectrum and the use of an Aluminum filter between the X-ray tube and the measurement window. Online X-ray fluorescence analysis of *P* entails some challenges due to the low characteristic energy of *P*, the phosphate slurry matrix and the online analysis mode. However, the outcomes of this study indicate that XRF is a promising technology to meet the requirement for digitalization of chemical analysis of phosphate products.

## Introduction

Phosphorus (*P*) is a fundamental constituent of plant life. It plays an important role in a range of plant metabolic functions and is one of the essential nutrients required for plant growth and development. Plants acquire all their *P* from fertilizers in the soil. The production of fertilizers begins with the transformation of phosphate rock into slurry (a mixture of dry phosphate and water) and the processing of the slurry using chemical processes. According to the World Resources Institute (WRI), there will be almost 10 billion people on the planet by 2050, the world will have to close a 56% gap between the amount of food available today and that required by 2050^1^. The world leaders in fertilizer production have a vital role to play in helping farmers around the world produce enough food. This role begins with increasing fertilizer production to meet growing demand, this is achievable through innovation and automation of fertilizer production lines. The digital transformation of industrial processes, also called industry 4.0 or the fourth industrial revolution, is a promising strategy to increase the production capacity of fertilizers. However, the transition to digitalization poses a real challenge for the phosphate processing field and more specifically in terms of quality control during production. Indeed, the quality control of the phosphate slurry is often related to the chemical analysis which are carried out in the laboratories using time-consuming conventional methods. Thus, chemical analysis is subject to significant delays, which complicates the rapid control of slurry treatment. Several methods exist for the determination of *P* during the phosphate slurry treatment process (e.g. gravimetric, titrimetric, photometric and colorimetric techniques)^2^. However, these methods require sample preparation that can take up to at 24 h^3–6^. A rapid *P* analysis system installed on the production line (online analysis) could overcome some challenges and allow rapid control of slurry treatment processes. Online analysis leads to several challenges such as different shapes of samples, moving samples, the possibility of a single pulse on a sample, variable composition, wet samples, the effect of physical parameters of the slurry, a dusty atmosphere, as well as heat and humidity^7^. As a result of all these challenges, very few experiences have been reported in the literature about on-line characterization of phosphate slurry. An online analyzer based on the neutron activation analysis (ANN) technique has been developed at the University of Florida to measure phosphate slurry. It is equipped with a ^241^Am/Be neutron source to measure the ${P}_{2}{O}_{5}$ content in the phosphate slurry. The direct measurement of ${P}_{2}{O}_{5}$ was not accurate due to interference from rare earth elements, in particular gadolinium (Gd) present in the phosphate slurry. However, the ${P}_{2}{O}_{5}$ content was estimated from the Ca analysis since most of the phosphate was present as fluorapatite (Ca_5_(PO_4_)_3_F)^8^. Despite the potential of the AAN technique to analyze phosphate slurry, the adoption of the technique remains expensive and presents a high risk associated with the use of neutron generators. The cost of procuring a neutron generator, with all its safety considerations, can therefore be prohibitive for a company^9^. A few experiments were carried out in order to verify the feasibility of using the Laser Induced Breakdown Spectroscopy (LIBS) technique for the online analysis of phosphate slurry. The quantification of *P* using its characteristic lines (253.7 nm and 255.5 nm) was not possible due to the low Signal to Noise Ratio (SNR). The characteristic line of *F* was used to estimate the ${P}_{2}{O}_{5}$ content assuming the presence of *P* in a single form which is the Fluorapatite Ca_5_(PO_4_)_3_F^10^. Online analysis of phosphate slurry using the LIBS technique cannot be considered the best choice due to its limitations such as splashing, surface turbulence and difficulties in getting reproducible samples due to sedimentation and the change of the lens-sample distance during the measurement^11–13^. Additionally, water can quench the laser plasma and lose the LIBS signal, resulting in poor sensitivity. The X-ray diffraction (XRD) technique was also used for the online analysis of the phosphate slurry. An online XRD analyzer developed by Mintek company from South Africa was used to analyze phosphate slurry in Florida. The maximum error in ${P}_{2}{O}_{5}$ measurements was 1.1%^14^. The XRD technique measures mineral content, not element content. It is essential to measure the content of *P* as an element when it is presented in several forms (Ca_5_(PO_4_)_3_F, H_3_PO_4_, Ca_5_(PO_4_)_3_Cl, etc.). Furthermore, due to the complexity of the setup, the implementation of an XRD analyzer remains prohibitively expensive.

This article presents the results of online XRF analysis of the phosphate slurry carried out using the Florida equipment (J&C Bachmann, Germany). Tests carried out in the J&C Bachmann laboratory have identified the optimal hardware configuration and measurement parameters for accurate online analysis of the ${P}_{2}{O}_{5}$ content in phosphate slurry. The online analysis of phosphate slurry using the XRF technique has never been reported in the literature and was far from being used in the phosphate industry. This is because of its poor performance when analysing light elements such as *P* with an X-ray characteristic energy of 2.01 keV. In contrast, the outcomes of this study are unexpected and confirm that XRF can be a promising technology for the phosphate processing industry. The use of the XRF technique in this application is an original and innovative proposal.

## Materials and methods

### Quality control of phosphate slurry

Phosphate slurry is a mixture of dry phosphate and water with a solids content of approximately 50%. It is the raw material to be exploited to produce fertilizers and other phosphate products, it is transported between production units by pipelines whose flow rates vary according to the production unit. Monitoring chemical composition is critical for controlling the phosphate slurry treatment process and improving the quality of phosphate products, which are frequently used as fertilizers. For example, quality control of fertilizers during production aims to meet other regulations related to the protection of the environment such as soil contamination. The customer requirements regulations are often related to chemical composition and vary depending on the specifications of the customer. Depending on the slurry treatment unit, samples are collected at varied time intervals ranging from 20 to 60 min. The samples are then delivered to the laboratory for examination based on solid content, particle size distribution, and concentration of some chemical components such as P_2_O_5_, CaO, SiO_2_, CO_2_, MgO, Cd, organic C, F, Cl, Al_2_O_3_, Fe_2_O_3_, K_2_O, Na_2_O, SO_3_, Zn, As, Cu and Mn. These chemical analyses are carried out in the laboratory using conventional analytical methods^15^. Monitoring the phosphorus or ${P}_{2}{O}_{5}$ concentration is critical to ensuring the quality of phosphate products. Indeed, the qualities of phosphate products are classified according to the ${P}_{2}{O}_{5}$ content or Bone Phosphate of Lime (BPL), a term often used in the phosphate industry to express the phosphate content of rock as tricalcium phosphate (${P}_{2}{O}_{5}$× 2.1853 = *BPL*).

## Samples and sample preparation

The phosphate slurry samples used in this study had a solid content between 50 and 55%, a particle size between 160 and 315 µm, a density of 1.47 g/ml, a P_2_O_5_ content between 13.5% and 18.5%, a CaO content between 23 and 27%, a SiO_2_ content between 3 and 7%, and an F content between 1.5 and 2.5%. These samples were prepared from dry phosphate rock extracted from five different layers; they were prepared to have slurry samples comparable to those that flowing along the production lines. According to the supplier, the ${P}_{2}{O}_{5}$ content in the slurry production lines varies between 13.5 and 16% depending on the production unit. The preparation of a slurry sample begins with the drying of the rock in an oven at 100 °C for 12 h. Then the water is added and the suspension is mixed for 5 min using a stirrer. To pump and analyze the samples, two distinct setups were constructed, one with a low flow rate of 1.3 l/min and vertical flow and the other with a high flow rate of 316 l/min and horizontal flow. In addition, two distinct sample sets were prepared to be evaluated with the two configurations. External laboratories measured the samples used in the present study to determine the actual ${P}_{2}{O}_{5}$ levels, each sample was analyzed by at least four laboratories. Table 1 summarizes the features of the 12 samples prepared for XRF analysis using the vertical flow setup, of which 8 samples were used to build the calibration curve. Table 2 summarizes the characteristics of the 10 samples that were prepared for XRF analysis using the horizontal flow setup, of which 5 samples were used to build the calibration curve.

**Table 1:** Characteristics of 12 samples prepared for XRF analysis using the vertical flow configuration.

	Sample ID	Particle size (µm)	Water content (%)	P_2_O_5_ (%)	Volume prepared (ml)
Samples prepared to build the calibration curve	C1/16	160	50	12.28 ± 0.07	70
	C1/18	160	50	13.53 ± 0.08	70
	C1/17	160	50	14.68 ± 0.11	70
	C2/15	160	50	15.38 ± 0.09	70
	C1/19	160	50	16.91 ± 0.13	70
	C2/18	160	50	18.24 ± 0.08	70
	C12/13	160	50	14.63 ± 0.08	70
	C10/13	160	50	14.58 ± 0.12	70
Test set	Mine 1	160	50	14.70 ± 1.41	900
	Mine 2	160	55	16.17 ± 1.55	800
	Mine 3	315	50	14.53 ± 1.16	900
	Mine 4	315	55	15.98 ± 1.28	800

**Table 2:** Characteristics of the 10 samples prepared for XRF analysis using the horizontal flow configuration.

	Sample ID	Particle size (µm)	Water content (%)	P_2_O_5_ (%)	Volume prepared (ml)
Samples prepared to build the calibration curve	Profil BG	315	50	14.00 ± 0.30	7000
	C2S_SB	315	50	15.58 ± 0.35	7000
	PBG_C0	315	50	14.50 ± 0.43	7000
	C2E_ID2	315	50	14.91 ± 0.54	7000
	C2SPBG_MIX	315	50	14.67 ± 0.03	7000
Test set	Layer 0	315	50	14.90 ± 0.61	7000
	Layer Sillon	315	50	14.72 ± 0.58	7000
	Layer 2 SUP	315	50	15.13 ± 0.42	7000
	Layer 2 EXP	315	50	14.80 ± 0.71	7000
	SBC0_MIX	315	50	14.90 ± 0.01	7000

### XRF setup for online analysis of phosphate slurry

**Figure 1:**
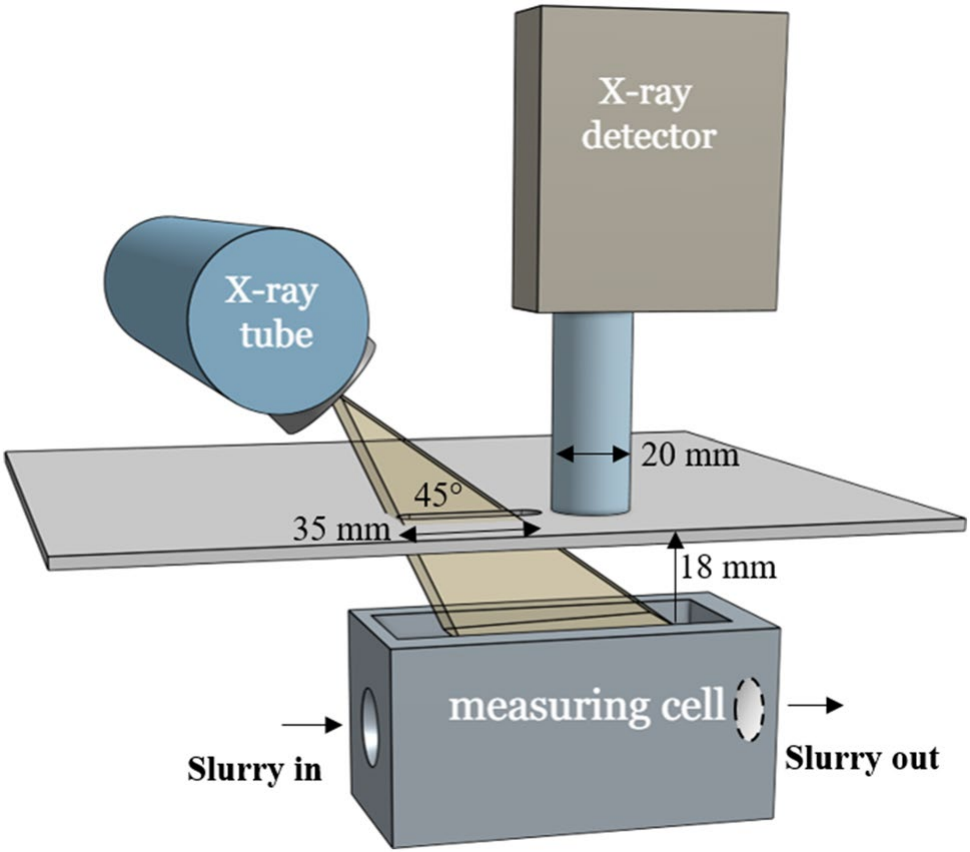
3D drawing of the XRF analyzer and the geometry of its main components.

**Figure 2:**
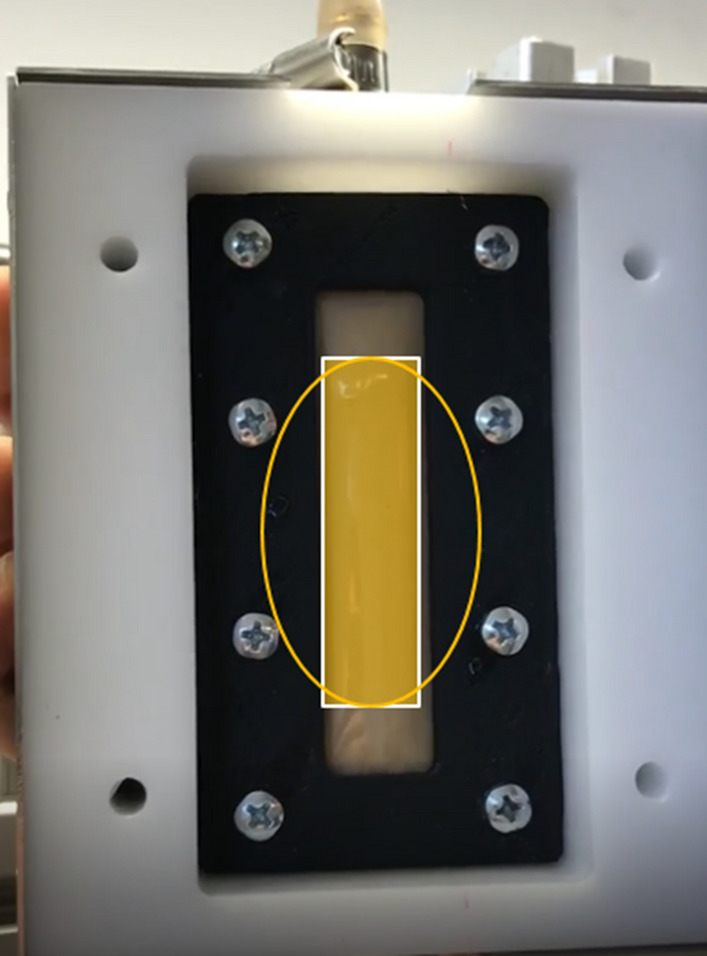
X-ray beam forms an elliptical spot of 52 × 65 mm on the surface of the measurement window, the surface of the irradiated sample is a rectangle of 20 × 65 mm.

The FLORIDA XRF device used for this study was developed by the German company J&C Bachmann, it includes a Molybdenum anode tube with a maximum voltage of 65 kV. A 3D drawing of the XRF analyzer is shown in the Fig. 1. The anode emission angle was adjusted to 45° for this application. The beams of X-ray emitted by the tube pass through the housing of the XRF analyzer through a rectangular opening of 35 × 8 mm and a minimum distance of 18 mm between the housing and the sample as shown in Fig. 1. The spot of the X-ray beams on the measuring cell is an eclipse of 52 × 65 mm while the surface of the irradiated sample is a rectangle of 20 × 65 mm as shown in Fig. 2. The detector is of the SDD type with a resolution of 122 eV FWHM and an active surface of 25 mm^2^ collimated at 17 mm^2^, it was placed at a distance of 18 mm from the protective film of the sample. The characteristic X-rays emitted by the atoms cross the XRF box through a circular opening with a diameter of 20 mm. The measurement cell has an 80 × 20 mm rectangular window protected by a plastic thin film. The continuously flowing slurry traverses the cell through a cylindrical chamber with a diameter of 20 mm. The MONACO software developed by the company J&C Bachmann was used to control the analyzer, choose the measurement parameters and build the calibration curves using a regression model. It offers numerous options, including normalizing the intensity of the peak $P\left(K\alpha \right)$ to the intensities of other elements to find the best calibration curve, recalculate peaks selected as potential features, extend available features by pair-wise multiplication and including their reciprocal, center data by fitting an intercept in the calibration model and group spectra by lab value entry. After creating a calibration curve, the ${P}_{2}{O}_{5}$ contents of unknown samples can be estimated.

### XRF setup for the analysis of a vertical flow

**Figure 3:**
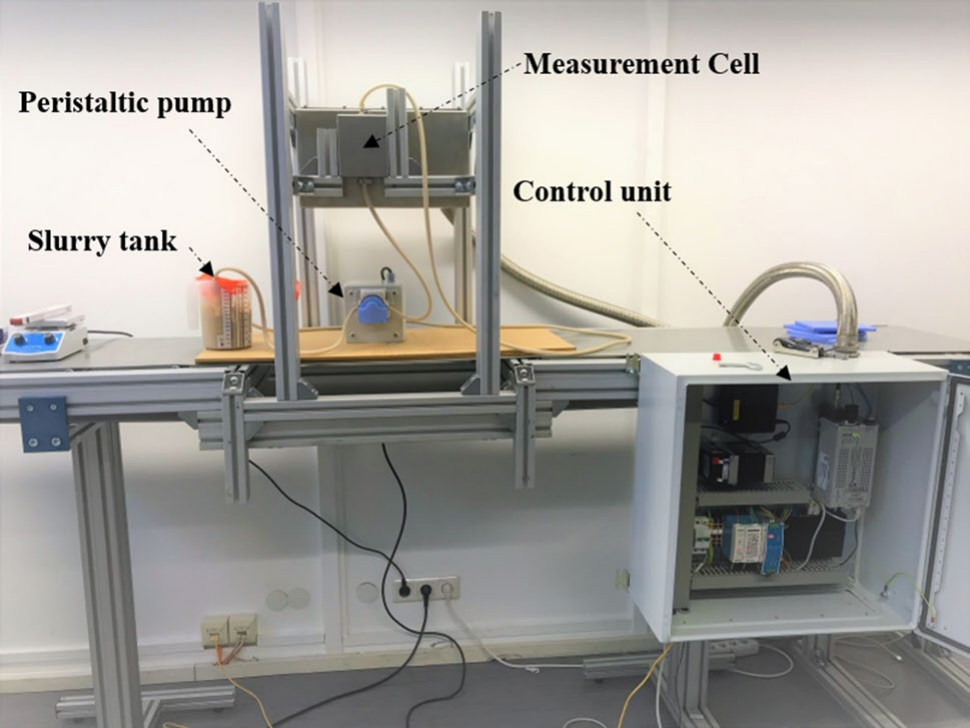
Setup built for the analysis of phosphate slurry in vertical flow.

A particular setup was built for the online analysis of phosphate slurry in vertical flow. The XRF analyzer was placed vertically and the flow in the measurement cell was always upwards. The pumping circuit includes a peristaltic pump with a maximum flow rate of 1.3 l/min, a slurry tank with a capacity of 2 L and a pipe with a diameter of 15 mm and a length of 3 m. The slurry traversing the measuring cell returns to the tank through the other end of the pipe. A stirrer was used to thoroughly mix and homogenize the slurry in the tank during the measurement time. Figure 3 shows a setup image. The analyses were performed using an excitation energy of 15 kV, a current of 1000 µA, a measurement time of 1 min per spectrum and 5 spectra collected per sample. The volumes of the 8 reference samples (about 70 ml per sample) were insufficient to pump them. To analyze these samples, one end of the cell was closed and slurry was added from the other end. Also, a drill with an improved drill bit was used to thoroughly homogenize and mix the slurry during the measurement. There was no segregation and the whole mixture was in motion during the measurement. Thus, it was possible to analyze these samples in online mode despite their small volumes. The 8 reference samples were analyzed to build the calibration curve. The volumes of the 4 validation samples (more than 800 ml per sample) were sufficient to pump them and perform a vertical flow analysis through the cell.

### XRF setup for the analysis of a horizontal flow

**Figure 4:**
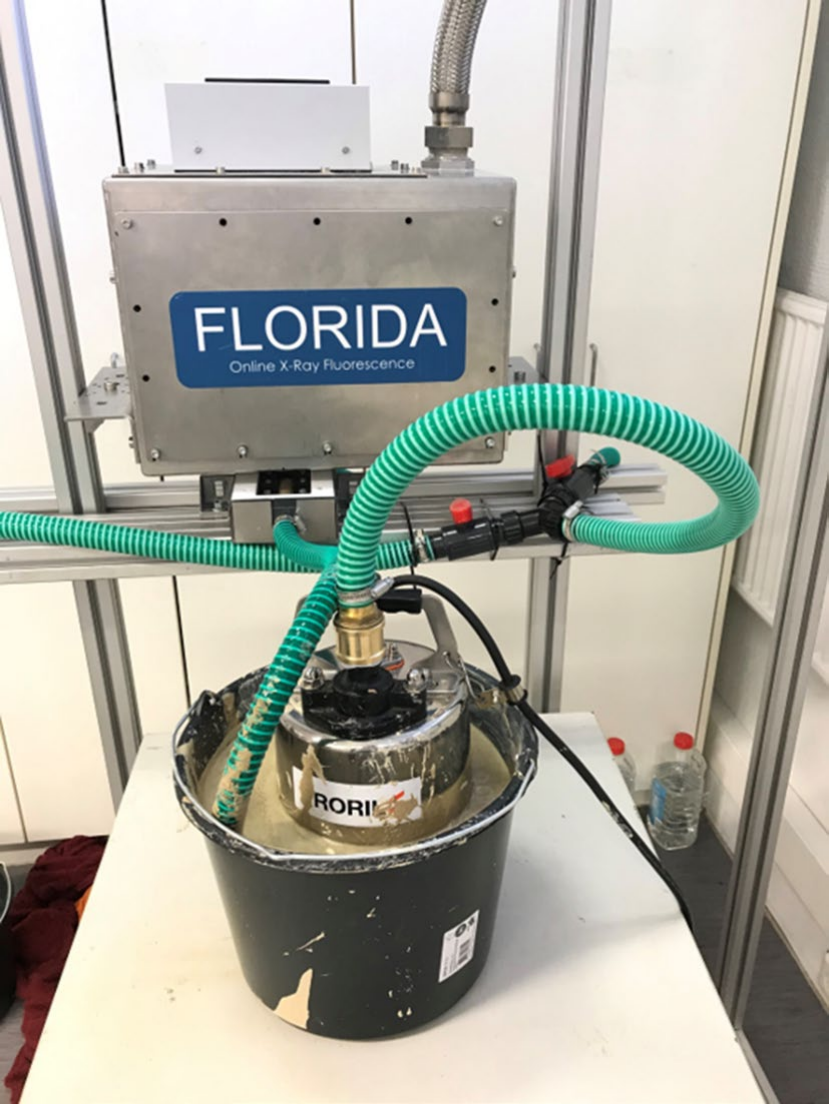
Setup built for the analysis of phosphate slurry in horizontal flow.

This setup is an improved version of the previous one, it was designed with the goal of analyzing a bigger volume of slurry at a higher flow rate. The XRF analyzer was turned 90 degrees to be able to analyze the horizontal flow from above. The SENTINO 750 pump (Habermann Aurum Pumpen, Germany) was used to pump at a flow rate of 316 l/min, it was immersed in a tank of phosphate slurry with a capacity of 12 L. Other pumps were tested, but due to the unique physical and mineralogical properties of phosphate slurry, they were unable to pump it. The slurry is well mixed during the measurement since it flows back to the tank with a high flow rate at the pipe outlet. As a result, it was unnecessary to use an agitator. Figure 4 depicts a setup image. The tests were performed with a 25 kV excitation energy, a current of 500 A, a measurement time of 60 s each spectrum, and a total of 10 spectra collected per sample. To create the calibration curve, five reference samples with a volume of 7 L each were assessed. In addition, a test set of five 7-L samples was analysed.

## Results and discussion

### XRF analysis using vertical flow setup

**Figure 5:**
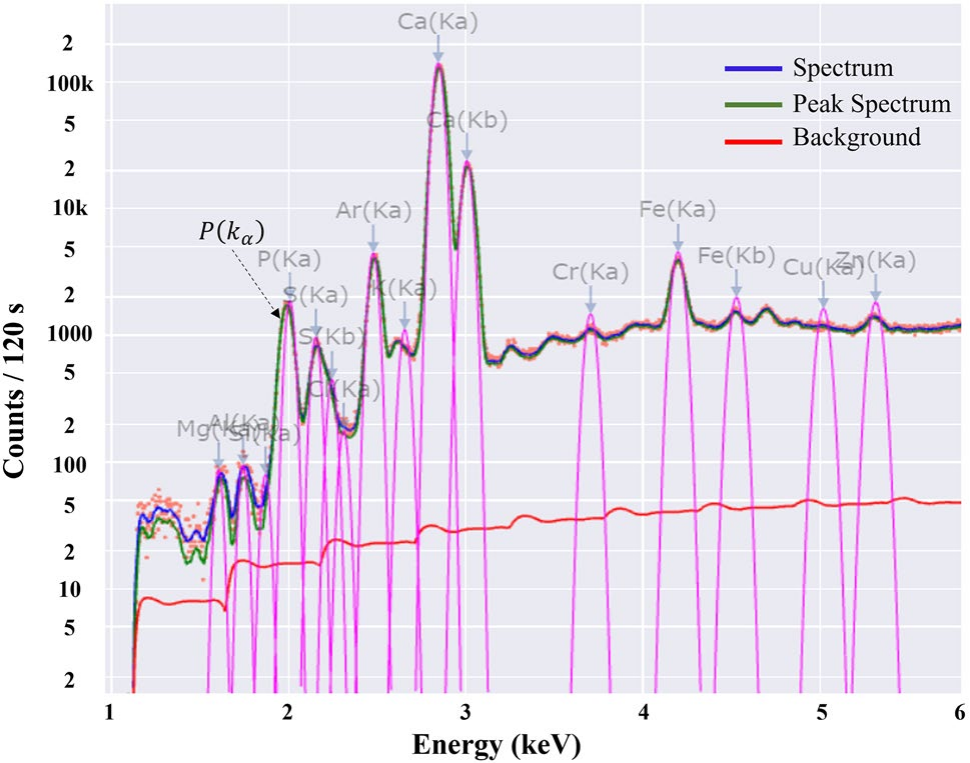
spectrum of a phosphate slurry sample analysed using the vertical flow XRF setup.

Prior to beginning the analysis of the samples prepared for this investigation, a sample of roughly one litre of phosphate slurry was pumped and tested numerous times to determine the best measurement settings. A good SNR of *P* was achieved by using an excitation energy of 15 kV, an excitation current of 1000 A, and a measuring time of 60 s. The resulting spectrum is depicted in Fig. 5, where the $P\left(K\alpha \right)$ characteristic peak of *P* is readily apparent with these measurement conditions and can be used to quantify ${P}_{2}{O}_{5}$.

**Figure 6:**
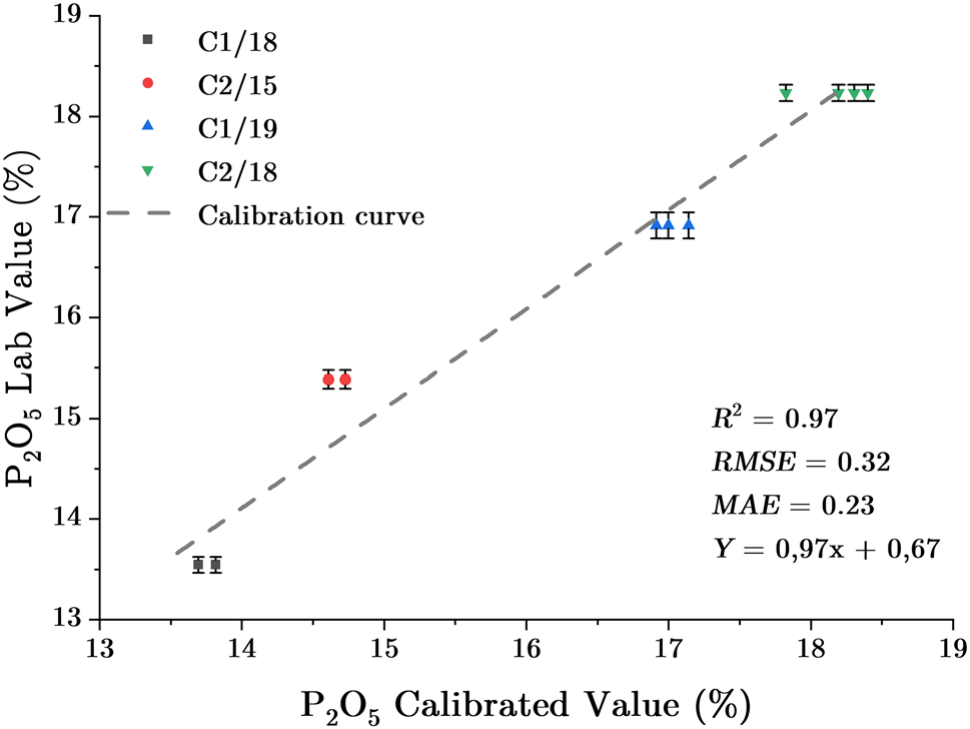
Calibration curve for online XRF analysis of P_2_O_5_ in phosphate slurry using the vertical flow setup.

The calibration curve was created by analysing the 8 reference samples. The software executes algorithms to figure out a correlation between the characteristic line intensity of selected elements and the ${P}_{2}{O}_{5}$ content of those samples. Also, it offers the option to normalize the intensity of $P\left(K\alpha \right)$ to other elements. There was no correlation between the intensities of $P\left(K\alpha \right)$ and the actual ${P}_{2}{O}_{5}$ concentrations of reference samples. However, by normalizing the intensity of $P\left(K\alpha \right)$, a better calibration curve was identified. This curve was created using four samples; adding more samples does not enhance it. The calibration curve with an *R*^*2*^ of 0.97, an *RMSE* of 0.32, and a *MAE* of 0.23 is shown in Fig. 6. The Y-axis shows ${P}_{2}{O}_{5}$ concentrations in % as determined by independent laboratories. The X-axis displays the concentration of ${P}_{2}{O}_{5}$ in % computed by the software based on the normalized intensities of the lines $P\left(K\alpha \right)$. This calibration curve covers a concentration range of ${P}_{2}{O}_{5}$ from 13.50 to 18.50%.

**Figure 7:**
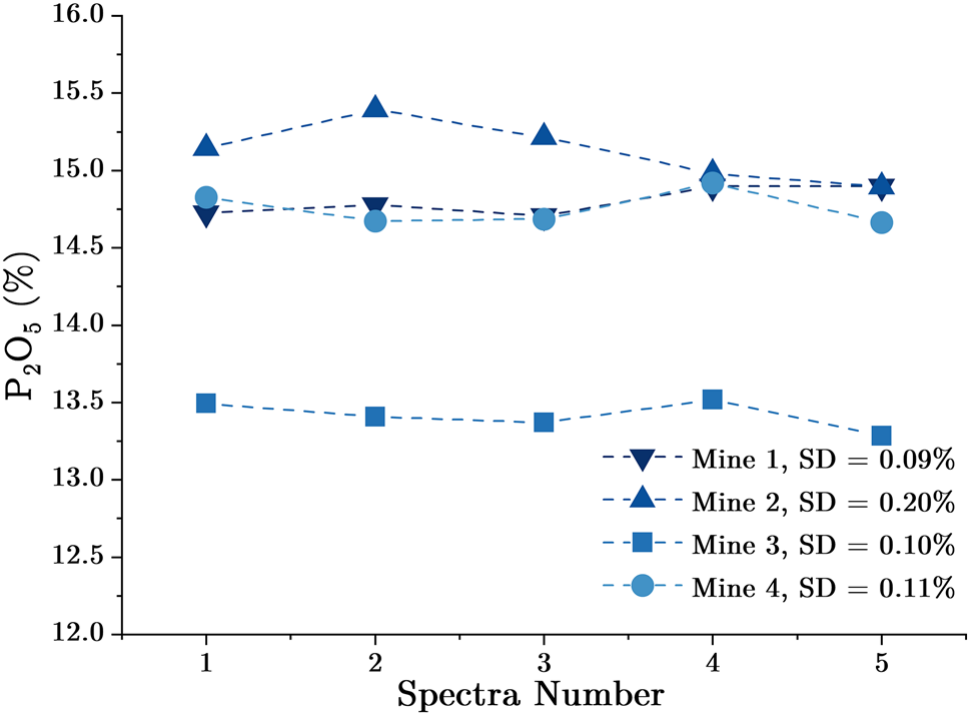
P_2_O_5_ concentrations calculated for each spectrum and each sample using the vertical flow XRF setup.

The test set contains four slurry samples of 800–900 ml each. They were analyzed in continuous flow using the setup shown in Fig. 3 and the same measurement parameters used to analyse the reference samples. Five spectra were collected for each sample and a ${P}_{2}{O}_{5}$ value was calculated for each spectrum using the calibration curve shown in Fig. 6. The calculated ${P}_{2}{O}_{5}$ values are given in Fig. 7, with the standard deviations for Mine 1, Mine 2, Mine 3, and Mine 4 being 0.09, 0.20, 0.10, and 0.11, respectively. These results demonstrate that the measurements taken with this setup are reliable. The actual ${P}_{2}{O}_{5}$ concentrations and the values measured using the vertical flow XRF setup are shown in Table 3. The minimal error observed is 0.10% for the Mine 1 sample, which has the same grain size of 160 m and solid content of 50% as the calibration set. The average absolute error using this setup was 0.87%, this value is considered acceptable if taking into consideration the differences between the physical matrix of the calibration set and the test set. The resulting error and calibration curve can be improved by constructing a better calibration. This is possible by analyzing several reference samples with a matrix comparable to that of the test set and volumes of at least 800 ml each sample in order to pump them. A second phase of testing is planned to implement these improvements, and an improved version of the setup is provided in the following section.

**Table 3:** Reference P_2_O_5_ concentrations and concentrations measured by the XRF vertical flow setup.

Sample ID	Actual concentration (%)	XRF measured concentration (%)	Absolute error (%)
Mine 1	14.70 ± 1.41	14.80 ± 0.09	0.10
Mine 2	16.17 ± 1.55	15.13 ± 0.20	1.04
Mine 3	14.53 ± 1.16	13.41 ± 0.10	1.12
Mine 4	15.98 ± 1.28	14.75 ± 0.11	1.23
MAE			0.87

### XRF analysis using horizontal flow Setup

**Figure 8:**
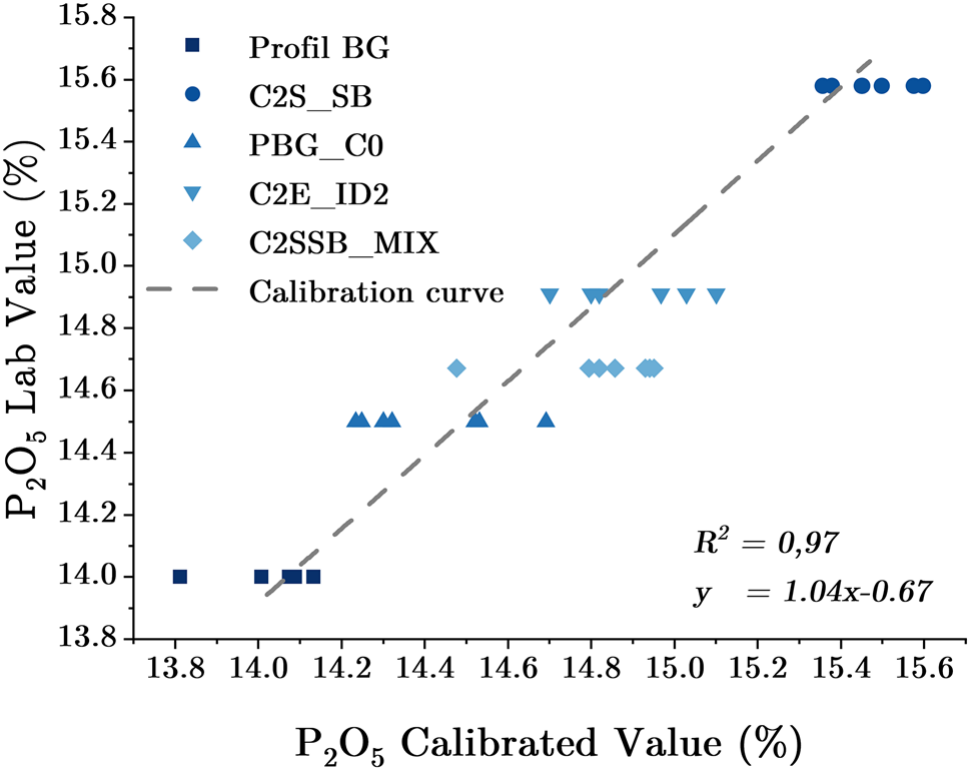
Calibration curve for online XRF analysis of P_2_O_5_ in phosphate slurry using the horizontal flow setup.

The experiments for this second setup were performed with an excitation energy of 25 kV, a current of 500 A, a measurement time of 60 s each spectrum, and ten spectra recorded per sample. This setup made it possible the analysis of samples with a volume of 7 L and a flow rate of 316 l/min. The five samples used to build the calibration curve have the same matrix as the test set. The calibration curve with an *R*^*2*^ of 0.97, an *RMSE* of 0.05, and a *MAE* of 0.03 is shown in Fig. 8. The Y-axis shows ${P}_{2}{O}_{5}$ concentrations in % as determined by independent laboratories. The X-axis displays the concentration of ${P}_{2}{O}_{5}$ in % computed by the software based on the calibration curve. This calibration curve covers a concentration range of ${P}_{2}{O}_{5}$ from 14.00 to 15.60%.

**Figure 9:**
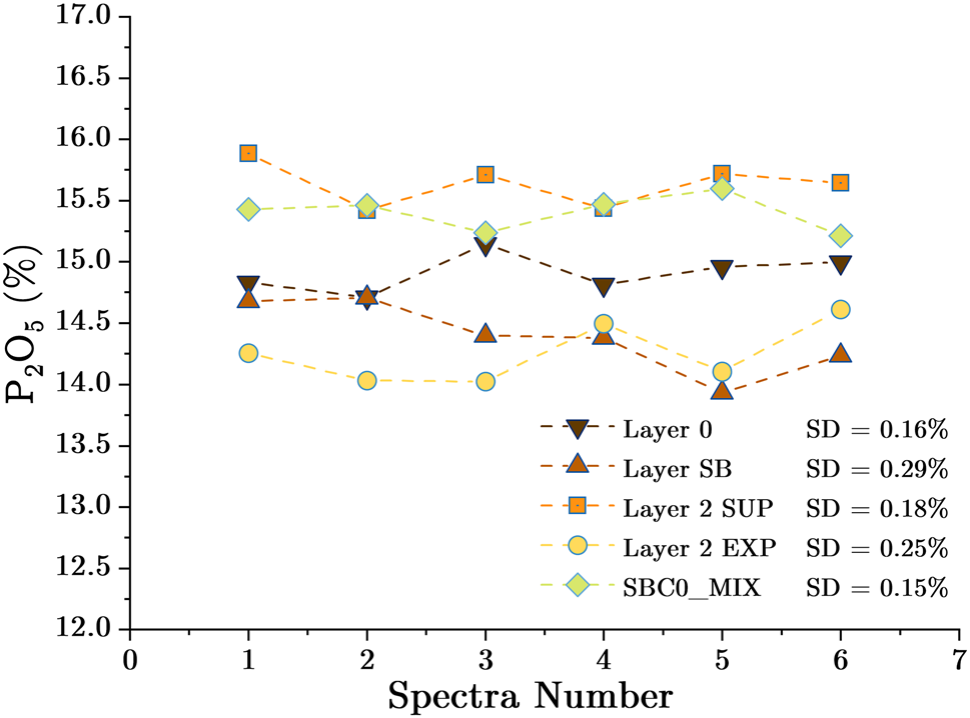
P_2_O_5_ concentrations calculated for each spectrum and each sample using the horizontal flow XRF setup.

The test set includes five slurry samples of 7 L each. They were analyzed in continuous flow using the setup shown in Fig. 4 and the same measurement parameters used to analyze the reference samples. For each sample, ten spectra were collected and a ${P}_{2}{O}_{5}$ value was calculated for each spectrum using the calibration curve shown in Fig. 8. As shown in Fig. 9, the standard deviations for Layer 0, Layer Sillon, Layer 2 SUP, Layer 2 EXP, and SBC0_MIX samples were 0.16, 0.29, 0.18, 0.25, and 0.15, respectively. Table 4 shows the reference ${P}_{2}{O}_{5}$ concentrations and the values measured using the vertical flow XRF setup. The minimum error achieved is 0.01% for the Layer 0 sample. The average absolute error has decreased from 0.87% in the previous setup to 0.38% in this setup.

**Table 4:** Reference P_2_O_5_ concentrations and concentrations measured by the XRF horizontal flow setup.

Sample ID	Actual concentration (%)	XRF measured concentration (%)	Absolute Error (%)
Layer 0	14.90 ± 0.61	14.91 ± 0.16	0.01
Layer Sillon	14.72 ± 0.58	14.39 ± 0.29	0.33
Layer 2 SUP	15.13 ± 0.42	15.64 ± 0.18	0.51
Layer 2 EXP	14.80 ± 0.71	14.25 ± 0.25	0.55
SBC0_MIX	14.90 ± 0.01	15.40 ± 0.15	0.50
MAE			0.38

**Figure 10:**
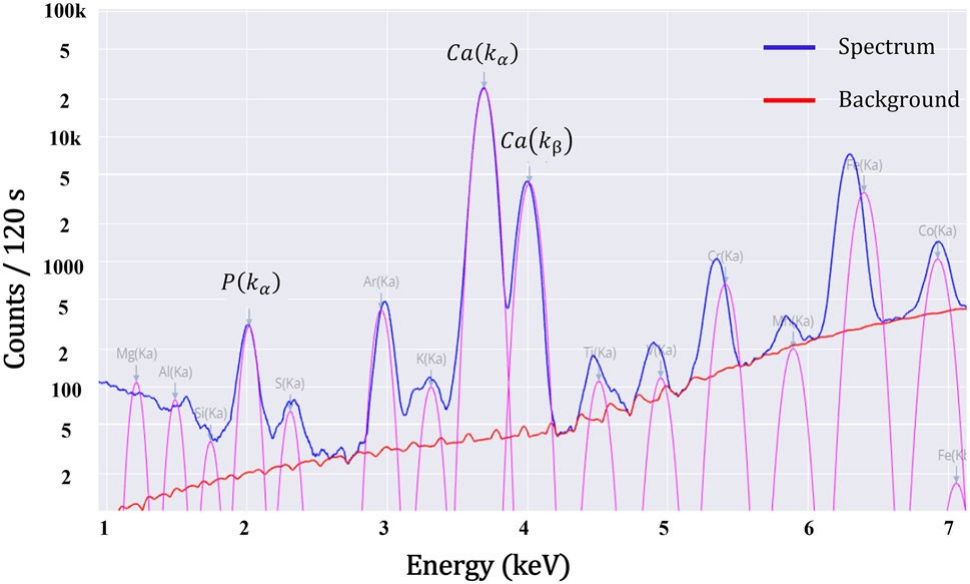
Spectrum of a phosphate slurry sample analysed using the horizontal flow setup.

The standard deviation figures observed with this setup are slightly higher than those of the prior one. This could be due to flow rate deviation and vibration of the protective film as a result of the usage of a very high flow rate. The analysis must be carried out under ideal conditions with a steady flow rate and the physical parameters of the slurry must not vary during the analysis. The rate of attenuation of the X-ray photons can also be affected by the vibration of the protective film (measurement window) or the variation in the distance between the material and the detector. When compared to the prior setup, the calibration curve for this one has been improved. Indeed, it was built by analysing more samples and normalizing the intensity of $P\left(K\alpha \right)$ peak, which is clearly visible in the spectrum, as shown in Fig. 10. This curve covers a concentration range of 14.00% to 15.60% ${P}_{2}{O}_{5}$ concentration, although it would be better to extend it to [13.50–16.00%] to cover some specific concentrations, which are rare.

The challenges of online XRF analysis of phosphate slurry are numerous. Indeed, the X-rays emitted by the *P* element have a low energy of 2.01 keV and can be quickly absorbed by water. Other obstacles involve the analysis of a complex matrix consisting of many elements and minerals, as well as the physical properties of the slurry and the analysis in online mode. However, the results achieved with this last setup demonstrated that the XRF technique is promising for online phosphate slurry analysis. Some improvements can be implemented to make the setup suitable for use in an industrial environment. Stabilizing the flow rate will improve the standard deviations of XRF measurements. The measurement parameters, the filters and the internal geometry of the analyzer can be optimized to reduce the attenuation of the X-rays characteristic of *P*.

### Optimal measurement parameters

**Figure 11:**
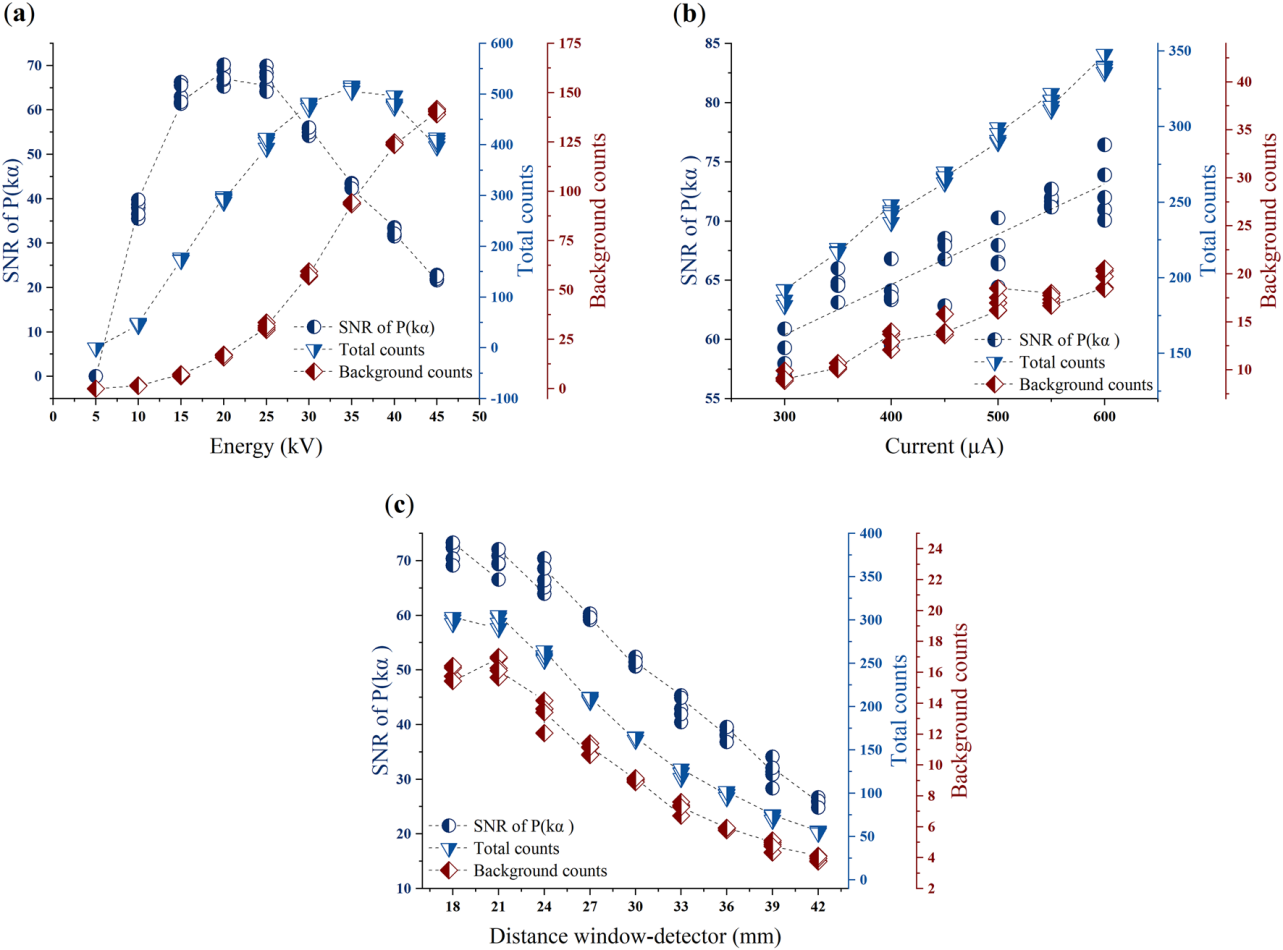
Signal to noise ration (SNR) of *P*(Kα) as a function of (**a**) excitation energy, (**b**) excitation current and (**c**) distance between sample and detector.

Excitation energy, current, filters, and measurement time can all be adjusted to make the device more sensitive to *P* measurement. The proper selection of an excitation energy for the measurement of phosphorus *P* allows for the avoidance of lines in the spectrum that can interfere with $P\left({k}_{\alpha }\right)$. To investigate the effect of excitation energy on the SNR of *P*, a phosphate sample was analyzed multiple times by varying the X-ray tube energy from 5 to 45 kV with a step of 5 kV, five spectra were collected for each measurement. The measurement time was 60 s per spectrum, the excitation current at 500 µA and the measurement cell was placed at the minimum distance from the detector. The signal to noise ratio (SNR) was computed using the following equation.

1 $$SNR=\frac{TC-BG}{\sqrt{BG}}$$

where, $TC$ is the total counts, which include signal and background, through the energy range of the $P\left(K\alpha \right)$ peak, $BG$ the background counts. The best SNR was attained using 20 kV or 25 kV excitation energy, as shown in Fig. 11a. When the excitation energy exceeds 25 kV, the background around $P\left(K\alpha \right)$ increases and the SNR drops linearly with the excitation energy. With an excitation energy of 5 kV, the peak $P\left(K\alpha \right)$ was not apparent, this low energy is quickly attenuated by water.

The current controls the intensity of the X-rays emitted by the generator and is primarily adjusted to optimize the signal level at the detector. The effect of excitation current on the SNR of $P\left(K\alpha \right)$ was investigated in this study. A phosphate sample was analyzed several times by varying the excitation current from 300 to 600 µA with a step of 50 µA, five spectra were collected for each measurement. The measurement time was 60 s per spectrum, the excitation energy was 25 kV and the measurement cell was placed at the minimum distance from the detector. The signal to noise ratio was calculated using Eq. (1). Figure 11b shows a linear correlation between the SNR of $P\left(K\alpha \right)$ and the excitation current, the best SNR was attained using a current of 600 µA. The intensity of $P\left(K\alpha \right)$ and the background evolve linearly according to the excitation current.

The distance between the sample and the detector can be chosen to reduce the attenuation of the characteristic X-rays of *P*. The minimum distance is 18 mm, although it can be increased by shifting the measurement cell from device box. The effect of this distance was studied by analyzing a phosphate sample several times and moving the measuring cell away from 18 to 42 mm with a step of 3 mm, five spectra were collected for each measurement. The measurement time for each spectrum was 60 s, the excitation energy was 25 kV, and the current was 500 A. Equation (1) was used to compute the SNR. The acquired results are illustrated in Fig. 11c, where the SNR drops as the sample-detector distance increases. With increasing distance, the background and intensity of $P\left(K\alpha \right)$ diminish. Because X-rays are absorbed by air and fade completely at a given distance, this outcome was expected. With a minimum distance of 18 mm, the best SNR was reached.

The background around $P\left(K\alpha \right)$ can be reduced by using a filter between the X-ray tube and the measurement window. The principal role of the primary source filter is to pass photons with energy high enough to excite the K-shell of *P*, and in the same time block energies that interfere with the fluorescence lines to be measured^16^. Two aluminum and copper filters were tested for this study, it was possible to reduce the background and to have a good visibility of $P\left(K\alpha \right)$ by using the aluminum filter. The SNR of $P\left(K\alpha \right)$ can also be improved by optimizing the measurement time. A longer measurements time improves the detection limit, the SNR and the measurement precision by reducing relative uncertainties^17^. Some tests were carried out as part of this study in order to optimize this time, and it was determined that a measurement of 60 s allowed for a good SNR. Measurements were taken for a longer duration, and the findings reveal no significant improvement in SNR.

## Conclusion

In this work, two XRF setups were built and optimized for online analysis of ${P}_{2}{O}_{5}$ content in phosphate slurry. Slurry samples identical to those circulating in the production line were studied, the average absolute error using the vertical flow and low flow setup was 0.87%. An improved version of this setup has been built to analyze a large volume of the sample in high flow and horizontal flow. The average absolute error recorded with this last setup was 0.38%. A slight increase in the standard deviation of the measurements was observed, this may be related to the flow rate deviation and the vibration of the protective film caused by the application of a high flow rate.

Several tests were carried out to determine the best configuration for online measurement of ${P}_{2}{O}_{5}$ in phosphate slurry using the horizontal flow setup. With excitation energies of 20 kV and 25 kV, an excitation current of 600 A, a minimal distance between the sample and the detector, a measurement time of 60 s per spectrum, and the employment of an aluminium filter between the X-ray tube and the measurement window, an excellent SNR of $P\left(K\alpha \right)$ was attained. The combination of all of these measurement parameters can increase the instrument's sensitivity to *P* measurement.

Despite the difficulties associated with using the XRF technique for phosphate slurry, the findings achieved with vertical flow setup demonstrated that the XRF technology is promising for this application. Some improvements can be implemented to make the setup suitable for use in an industrial environment. The flow rate stabilization will enhance the standard deviation of the XRF measurements, and the real-time acquisition of particle size and solids content data will contribute to correct the measured concentration and improve the online analysis of ${P}_{2}{O}_{5}$.

## Data Availability

The datasets used and/or analysed during the current study available from the corresponding author on reasonable request.
