# Author Correction: XRF online analyzer for measurements of P_2_O_5_ content in phosphate slurry

**DOI:** 10.1038/s41598-023-46582-2

**Published:** 2023-11-13

**Authors:** Ismail Ben Amar, Andrew Thomas, Claus Bachmann, Anass Hafnaoui, Hafid Griguer, Amine Miled, Younès Messaddeq

**Affiliations:** 1 Department of Electrical and Computer Engineering, Université Laval, Quebec City, QC Canada; 2 Digital Innovation Center of Excellence DICE, Mohammed VI Polytechnic University UM6P, Ben Guerir, Morocco; 3 J&C Bachmann GmbH, Pfinztal, Germany; 4 OCP Group S.A, Jorf Lasfar, El Jadida, Morocco; 5 Center for Optics, Photonics, and Lasers, Université Laval, Quebec City, QC Canada

Correction to: *Scientific Reports* 10.1038/s41598-023-45181-5, published online 20 October 2023

The original version of this Article contained an error in the name of Anass Hafnaoui, which was incorrectly given as Anass Hafnoaui.

The original Article has been corrected.

